# Ferroptosis inhibitor alleviates Radiation-induced lung fibrosis (RILF) via down-regulation of TGF-β1

**DOI:** 10.1186/s12950-019-0216-0

**Published:** 2019-05-29

**Authors:** Xuan Li, Lijie Duan, Sujuan Yuan, Xibing Zhuang, Tiankui Qiao, Jian He

**Affiliations:** 1 Department of Radiation Oncology, Zhongshan Hospital, Fudan University, 180 Fenglin Road, Shanghai, 200032 China; 2 Department of Radiation Oncology, Jinshan Hospital, Fudan University, 1508 Longhang Road, Shanghai, 201508 China; 3 Department of Neurology, Jinshan Hospital, Fudan University, 1508 Longhang Road, Shanghai, 201508 China

**Keywords:** Radiation-induced lung fibrosis, Ferroptosis, Ferroptosis inhibitor, TGF-β1, ROS, Nrf2

## Abstract

**Background:**

Radiation-induced lung fibrosis (RILF) is a severe and life-threatening complication of thoracic radiotherapy. Cell death is the key issue in RILF. Ferroptosis is a form programmed cell death implicated in the pathologies of inflammation. This study aimed to investigate the role of ferroptosis in RILF, and the effectiveness and the potential underlying mechanism of ferroptosis inhibitor on RILF.

**Methods:**

Immunofluorescence, western blot and RT-PCR assays were performed to examine the ferroptosis maker glutathione peroxidase 4 (GPX4) in a mice RILF model. The lung tissue sections were stained with hematoxylin and eosin (H&E), Masson trichrome staining and Sirius-Red staining to evaluate the histopathological changes in RILF mice. Reactive oxygen species (ROS) and hydroxyproline (HYP) in lungs were measured by the relevant kits. The serum levels of inflammatory cytokines (TNF-α, IL-6, IL-10, and TGF-β1) were measured with Elisa. The protein and mRNA levels of GPX4, nuclear factor (erythroid-derived 2)-like 2 (Nrf2), hemeoxygenase-1 (HO1) and quinone oxidoreductase 1 (NQO1) in lungs were examined by western blot and RT-PCR.

**Results:**

GPX4 levels of the irradiated lungs were significantly down-regulated than the groups with no irradiation, and the ferroptosis inhibitor, liproxstatin-1, increased GPX4 levels significantly in RILF mice. Treatment with liproxstatin-1 lowered the Szapiel and Ashcroft scores significantly, down-regulated the levels of ROS and HYP in lungs and reduced the serum inflammatory cytokines levels in RILF mice. The protein and the mRNA levels of Nrf2, HO1 and NQO1 were up-regulated by liproxsratin-1 in RILF.

**Conclusions:**

Our data suggested that ferroptosis played a critical role in RILF, ferroptosis inhibitor liproxstatin-1 alleviated RILF via down-regulation of TGF-β1 by the activation of Nrf2 pathway. The effectiveness of ferroptosis inhibition on RILF provides a novel therapeutic target for RILF.

## Background

Radiation-induced lung fibrosis (RILF) is a severe and life-threatening complication in thoracic cancer patients with radiotherapy [1, 2]. Symptomatic RILF occurs several months after initial radiation therapy and can persist for up to 2 years [3, 4]. RILF occurs in 13–37% of lung cancer patients undergoing curative radiotherapy [5]. However, no effective strategy to manage for RILF has been identified [4]. RILF involves a cascade of inflammatory events following ionizing radiation (IR) [6, 7]. A burst of inflammatory cytokines induced by IR stimulates the progression of RILF, and among these cytokines, transforming growth factor-β1 (TGF-β1) plays a critical role in the pathogenesis and development of RILF [8, 9]. Excessive Reactive oxygen species (ROS), induced by IR, were reported to induce pulmonary injury, inflammation and fibrosis in RILF, and ROS-induced oxidative damage is considered to be a critical origin of inflammatory events involved in RILF [10, 11].

Ferroptosis is a recently recognized form of regulated cell death that involves iron accumulation and lipid peroxidation, the death phenotype of which is distinct from other cell mortalities, such as apoptosis and necroptosis [12]. The accumulation of ROS plays a key role in inducing Ferroptosis [13]. Glutathione peroxidase 4 (GPX4), a lipid repair enzyme, is the central regulator of ferroptosis [14]. Ferroptosis has been reported to be involved in many diseases, such as cancer, ischemia-reperfusion injury, myocardial infarction and many neurological diseases [15]. The major factor that triggers ferroptosis is ROS accumulation [16], which is also the major trigger to induce inflammatory cytokines in RILF. Additionally, ferroptosis Inhibitor has been shown to have anti-inflammation effects by suppressing the expression of the inflammatory cytokines including TGF-β1 in contusion spinal cord injury [17]. Therefore, we speculate that ferroptosis may occur in RILF, and the inhibition of ferroptosis may alleviate the RILF via down-regulation of TGF-β1 .

Nuclear factor (erythroid-derived 2)-like 2 (Nrf2) plays a key role in regulating antioxidant molecules in cells [18]. Enhancing NRF2 activity could prevent many diseases in which oxidative and inflammatory stress are crucial for pathogenesis [19, 20]. It is well known that activation of the Nrf2 signaling protects cells against radiation-induced oxidative damage [21, 22]. At the same time, the activation of the Nrf2 signaling could down-regulate TGF-β1, and alleviates the fibrosis diseases [23–25]. Nrf2 signaling also plays a protective role against ferroptosis in many diseases [26, 27]. Therefore, Nrf2 pathway might be involved in this process of inhibition of ferroptosis on RILF.

In this study, we investigated the role of ferroptosis in RILF mice model, and explored the therapeutic effects and the underlying mechanism of ferroptosis inhibitor on RILF. Our data suggested that ferroptosis played a critical role in RILF, and ferroptosis inhibitor liproxstatin-1 alleviated RILF via down-regulation of TGF-β1 by the activation of Nrf2 pathway. The insights gained from this research advanced our understanding of the cell death pathways in RILF and introduced a new therapeutic approach for treating RILF.

## Materials and methods

### Animals model and experimental design

Female C57BL/6 mice weighing 20 ± 2 g were, 4-6 weeks old, obtained from the Jiesijie Laboratory Animal Company (Shanghai, China) (Medical Experimental Animal Number SCXK 2013–0006). Liproxstatin-1 was purchased from MCE (New Jersey, USA). According to the manufacturer’s instructions, compounded the liproxstain-1 solution as follows, dissolved 10 mg liproxstain-1 with PBS to 20 ml (with the concentration of lipproxstain-1 0.5 mg/ml). Mice were randomly assigned to 4 groups as follows: Control group, IR group (received radiation), IR + Lip-1 group (injected with liproxstatin-1 and received radiation) and Lip-1 group (injected with liproxstatin-1 and no radiation). All experimental procedures and protocols were approved by the Ethics Committee of Jinshan Hospital Affiliated to Fudan University (Jinshan Ethics-2018-05). Mice were anesthetized and received a single of 15 Gy to the thorax. The beam was 6 MV X-ray at a dose rate of 2.0 Gy/min. The source-surface diatance (SSD) was 100 cm. The radiation field was 2 cm × 2 cm. Non-irradiated mice underwent the same procedure but were not exposed to the radiation. The mice were sacrificed and samples were collected at 20 weeks after radiation. The liproxstatin-1 (10 mg/kg) was administered by intraperitoneally injection 30 min post radiation, and was injected once a day until day 30 post radiation [28]. All aspects of testing and data analysis employed a blinded design.

### Serum collection and tissue isolation

Mice were sacrificed at indicated time. Retro-ocular artery blood was collected and centrifuged at 5000 rpm for 5 min and another 5 min at 3000 rpm. The serum was collected and then kept it at − 20 °C for analysis of cytokines. The upper left lung lobes was fixed in 4% paraformaldehyde and then embedded in paraffin for analysis of histopathology. The rest lung tissues were snap-frozen in liquid nitrogen and used for analysis of immunofluorescence of GPX4 and measurement of ROS content, hypdroxyproline (HYP) content and the protein and mRNA levels of GPX4, Nrf2, hemeoxygenase-1 (HO1) and quinone oxidoreductase 1 (NQO1).

### Histopathological analysis

The left lung specimens were embedded in paraffin and cut into 4 μm-thick slices. The tissue sections were stained with hematoxylin and eosin (H&E), Masson trichrome staining (KeyGEN BioTECH, Nanjing, China) and Sirius-Red staining (KeyGEN BioTECH, Nanjing, China), respectively. The Szapiel score was used to evaluate the pulmonary fibrosis stained with H&E [29]. The pulmonary fibrosis stained by Masson trichrome and Sirius-Red staining were evaluated according to the Ashcroft score [30]. Two blind independent pathologists performed the analysis. A mean score of Szapiel or Ashcroft for each mice was used for statistical analysis.

### Immunofluorescence staining analysis

Fixed cryosections (10-μm-thick) with 4% polyformaldehyde stationary solution at room temperature for 20 min, and then washed them in PBS for 3 min three times. Then, incubated the sections with a blocking solution (50-100 μl normal goat serum) at room for 20 min. Next, incubated the sections with incubated with anti-GPX4 antibody (14432-AP, Proteintech, USA) overnight at 4 °C. Washed the sections for 3 min with PBS three times, and then incubated them with the secondary antibodies (KGAA26, KeyGEN BioTECH, Nanjing, China) for 60 min at room temperature. Then washed the nuclei for 3 times and counterstained the nuclei with DAPI (KeyGEN BioTECH, Nanjing, China) for 5 min. Finally, a drop of anti-fade mounting medium was placed on each slide. The immunofluorescence imaging was visualized using Olympus fluorescence microscope equipped with a digital camera system (BX43, Japan). Image J software (NIH) was used to assess the intensity of GPX4 positive area for quantifying GPX4 expression.

### Measurements of ROS content and HYP content

Reactive oxygen species (ROS) determination kit (Biyuntian Institute of Biotechnology, China) was used to measure ROS content in lung tissues in mice. Right lung tissues were sheared to homogenates with 0.9% sodium chloride solution (mg: ml = 1:9), and then the homogenates were centrifuged (2500 rpm/min, 10 min). The supernatants were incubated with the fluorescent probe (DCFH-DA, Biyuntian, China) for 60 min at room temperature. The fluorescence values were measured by the multifunctional enzyme label instrument (MD Spectramac M3, USA). The results were expressed by fluorescence intensity / mg protein.

A hydroxyproline (HYP) assay kit (KeyGEN BioTECH, Nanjing, China) was used to measure the HYP content of lung tissue samples. Homogenized the lung tissue in trichloroacetic acid. Then washed the pellets from the homogenates with distilled water and hydrolyzed them with HCl. After neutralizing the hydrolysates with NaOH, added the chloramines-T and dimethylaminobenzaldehyde consecutively. Subsequently, evaluated the hydroxyproline content in lung tissue by recording the absorbance at 550 nm. The results were expressed as μg hydroxyproline/ mg wet lung.

### Enzyme-linked immunosorbent assay

The Elisa kits (KeyGEN BioTECH, Nanjing, China) were used to measure necrosis factor-α (TNF-α), interleukin-6 (IL-6), interleukin-10 (IL-10), and TGF-β1 levels in mice serum according to the manufacture instructions.

### Western blot analysis

Lung tissues were homogenized and incubated in lysis buffer containing a protease inhibitor cocktail. The protein concentrations were measured using a BCA kit (KGA902, KeyGEN BioTECH, Nanjing, China), and proteins were then denatured at 100 °C for 5 min. Proteins were loaded onto a 10% SDS-PAGE gel and transferred to a polyvinylidene fluoride (PVDF) membrane. Blots were blocked at room temperature for 1 h and then incubated with primary antibodies at 4 °C overnight. The details of primary antibodies used in this paper were shown as follows: rabbit anti-GPX4 (14432–1-ap, Proteintech, 1:500), rabbit anti-Nrf2 (ab31163, abcam, 1:500), mouse anti-HO1 (sc-136,960, santa, 1:200), and mouse anti-NQO1 (sc-32,793, santa, 1:200), rabbit anti-GAPDH (KGAA002, KeyGEN BioTECH, China, 1:10000). After washing, blots were incubated with fluorescence-labeled secondary antibodies.

### Real time PCR analysis

Total RNA samples were isolated from the right lung tissues with Trizol (15596–026, Invitrogen USA). The first strand cDNA was synthesized with the reverse transcription kit (RR036B, TaKaRa, Japan). Relative mRNA expressions of the Nrf2, HO1, and NQO1 gens were assayed by quantitative real time PCR kit (RR036B, TaKaRa, Japan) with GAPDH as internal control. The PCR primer sequences for the gens were showed as follows:

GPX4, forward primer: TAAGAACGGCTGCGTGGTGAAG, reverse primer: AGAGATAGCACGGCAGGTCCTT;

Nrf2, forward primer: TCAGGCCCAGTCCCTCAATA, reverse primer: TCCTGCCAAACTTGCTCCAT;

HO1, forward primer: GAAATCATCCCTTGCACGCC, reverse primer: CCTGACAGGTCACCCAGGTA;

NQO1, forward primer: CATTGCAGTGGTTTGGGGTG, reverse primer: TCTGGAAAGGACCGTTGTCG;

GAPDH, forward primer: AAGGTCGGTGTGAACGGATT, reverse primer: TGAGTGGAGTCATACTGGAACAT.

### Statistical analysis

Analysis was carried out through SPSS 21.0 (IBM Corporation, Armonk, NY, USA). Results were presented as mean ± SD. The comparisons for multiple groups were carried out by one-way ANOVA followed by a Bonferroni correction. *P* < 0.05 was considered statistically significant.

## Results

### Ferroptosis inhibitor up-regulated GPX4 levels of the lungs in RILF

**Fig. 1:**
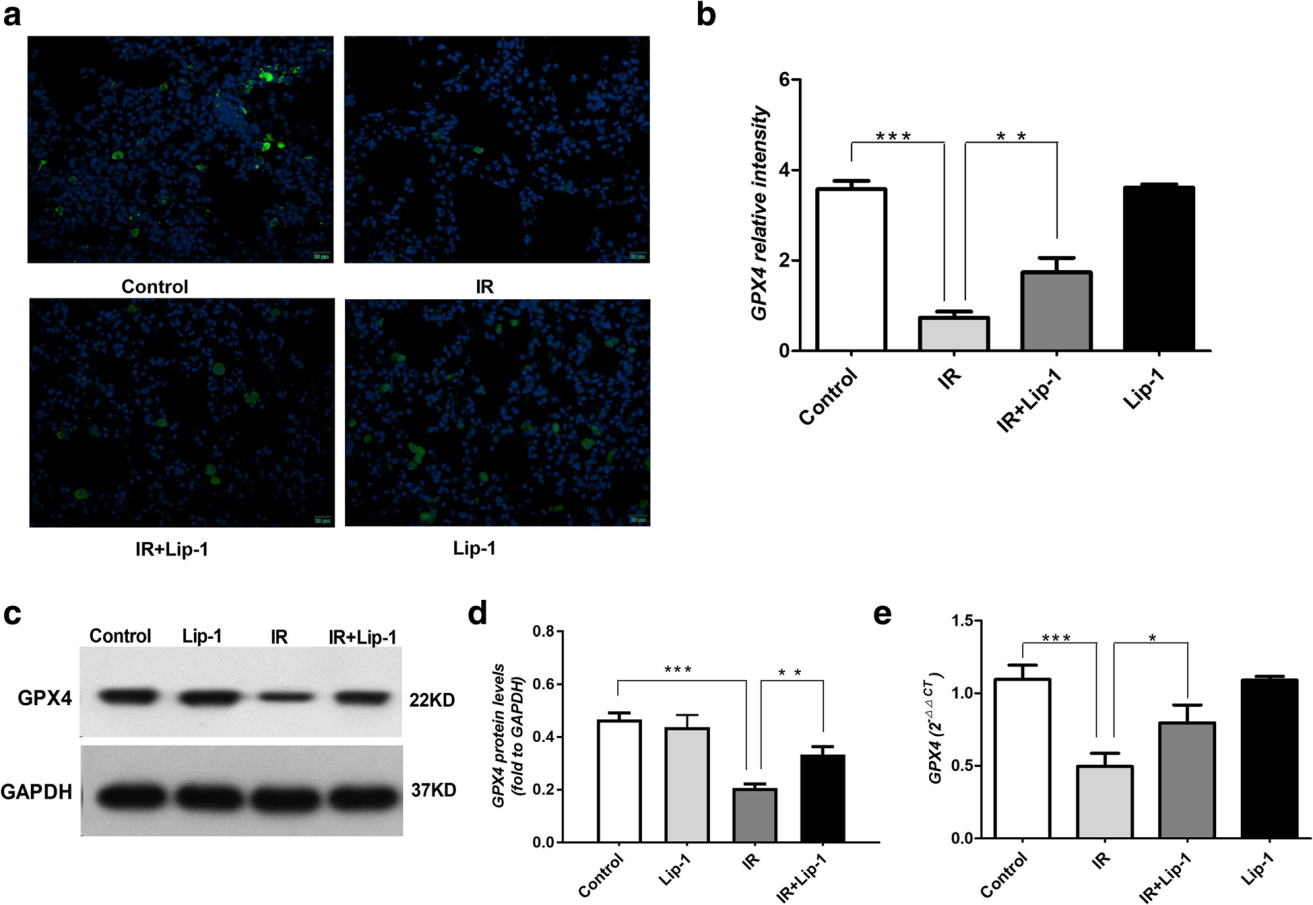
The effect of lipoxstatin-1 on GPX4 expressions following RILF. **a** Representative fluorescence micrographs of GPX4 staining in lungs. Scale bar is 20 μm. **b** Quantification of GPX4 expressions. **c** The protein levels of GPX4 in lungs were evaluated by western blotting. **d** Quantification of the protein levels of GPX4 in lungs. **e** Quantification of the mRNA levels of GPX4 in lungs were evaluated by Real time PCR. (Data shown as mean ± SD, one-way ANOVA followed by a Bonferroni correction, * *P* < 0.05, ** *P* < 0.01, *** *P* <0.001, *n* = 6)

GPX4 is the central regulator of ferroptosis, and the decline of GPX4 is often used as a marker of ferroptosis [12, 14, 28]. In this paper, we detected the expression of GPX4 of the irradiated lungs with immunofluorescence staining, western blot and real time PCR (Fig. 1a-e). By the analysis of the immunofluorescence staining, the GPX4 levels of the irradiated lungs were significantly down-regulated than the groups with no irradiation (Fig. 1b, *p* < 0.001). After treatment with liproxstatin-1, the levels of GPX4 were up-regulated (Fig. 1b, *p* < 0.01). The analysis of western blot and real time PCR also showed that the protein and mRNA levels of GPX4 in the irradiated lungs were significantly down-regulated (Fig. 1d, e, *p* < 0.001), and the administration of liproxstatin-1 up-regulated GPX4 levels in RILF mice (Fig. 1d, *p* < 0.01 and Fig. 1e, *p* < 0.05). The results supported that ferroptosis occurred in the process of RILF.

### Ferroptosis inhibitor mitigated the histological alteration in RILF mice

**Fig. 2:**
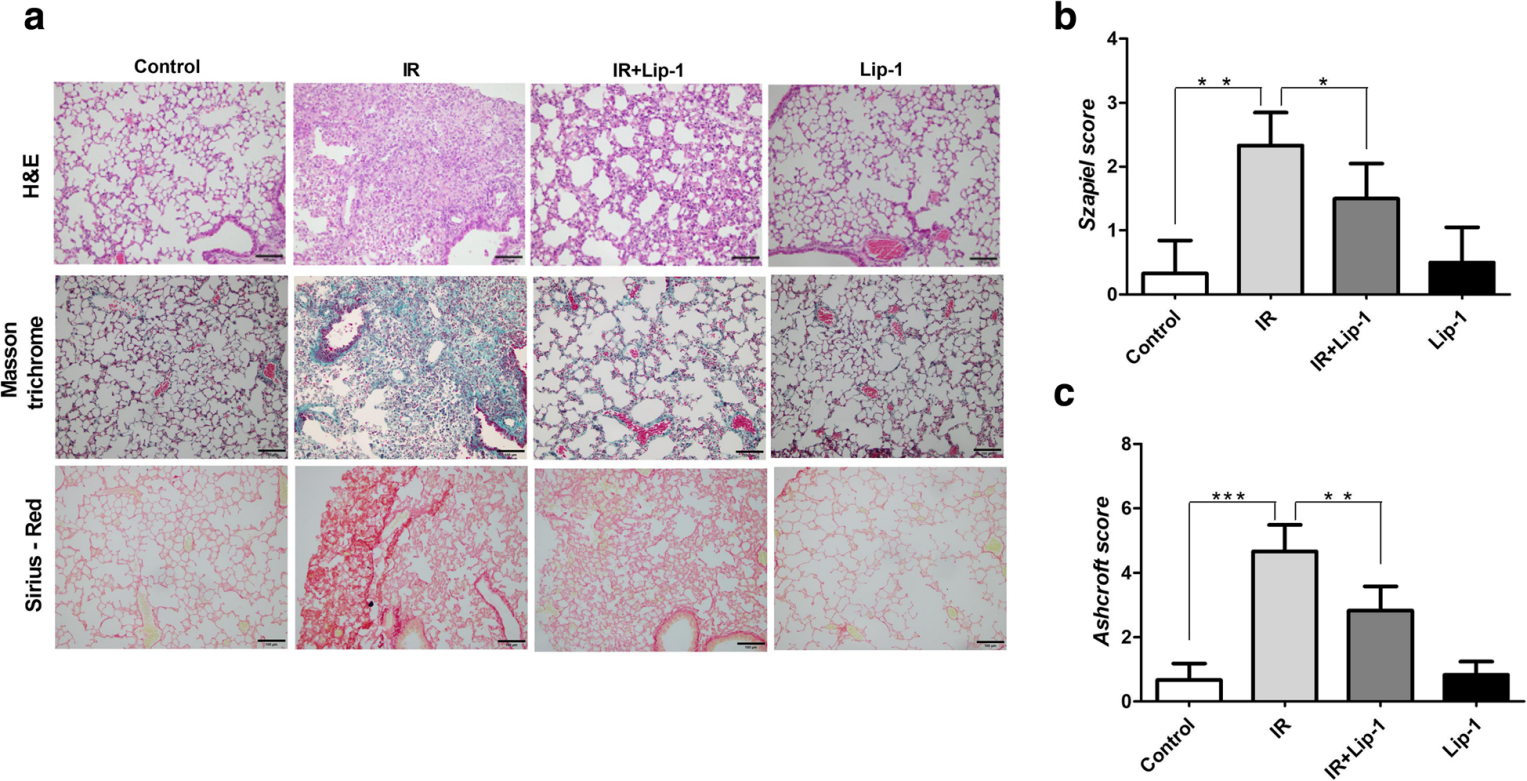
The effect of liproxstatin-1 on histology outcome following RILF. **A** H&E staining, Masson trichrome staining and Sirius-Red staining were used to evaluate IR-induced fibrosis of the lung tissues. Scale bar: 100 μm. The histopathological slides were evaluated using a semiquantitative scoring method. **b** The Szapiel score was used to evaluate the pulmonary fibrosis stained with H&E. **c** The pulmonary fibrosis stained by Masson trichrome and Sirius-Red staining were evaluated according to the Ashcroft score. (Data shown as mean ± SD, one-way ANOVA followed by a Bonferroni correction, * *P* < 0.05, ** *P* < 0.01, *** *P* <0.001, *n* = 6)

Extensive sub-pleural collagen accumulations were observed in sections of the lungs in irradiated group, and these pathologic changes were fewer in that of irradiated along with liproxstatin-1 mice (Fig. 2a). The Szapiel scores for H&E staining (Fig. 2b, *p* < 0.05) and the Ashcroft scores for Masson-trichrome staining and Sirius-Red staining (Fig. 2c, *p* < 0.01) of the irradiated mice treated with liproxstatin-1 were lower than those of the irradiated mice significantly. The results supported that administration of liproxstatin-1 resulted in inhibiting the collagen deposition in RILF mice.

### Ferroptosis inhibitor decreased the contents of HYP in RILF mice

**Fig. 3:**
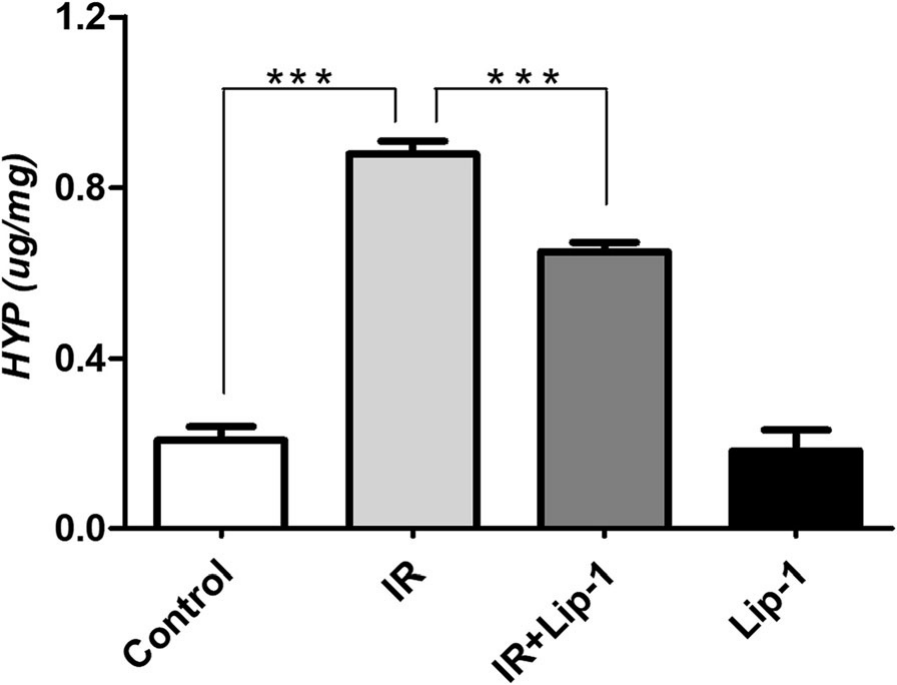
The effect of lipoxstatin-1 on HYP content in RILF. (Data shown as mean ± SD, one-way ANOVA followed by a Bonferroni correction, *** *P* < 0.001, *n* = 6)

To further determine the anti-fibrotic effect of liproxstatin-1 on RILF mice, HYP contents in mouse lungs were measured. HYP is characteristic of collagen fiber, and it is commonly used to reflect the degree of pulmonary fibrosis [31]. The HYP contents of the lungs increased in RILF mice, and this was suppressed by liproxstatin-1 treatment significantly (Fig. 3, *p* < 0.001). This result was consistent with the results observed in the pathological sections, which suggested that ferroptosis inhibitor could reduce collagen deposition in irradiated lungs, and alleviated RILF in mice.

### Ferroptosis inhibitor reduced inflammatory cytokines and ROS in RILF mice

**Fig. 4:**
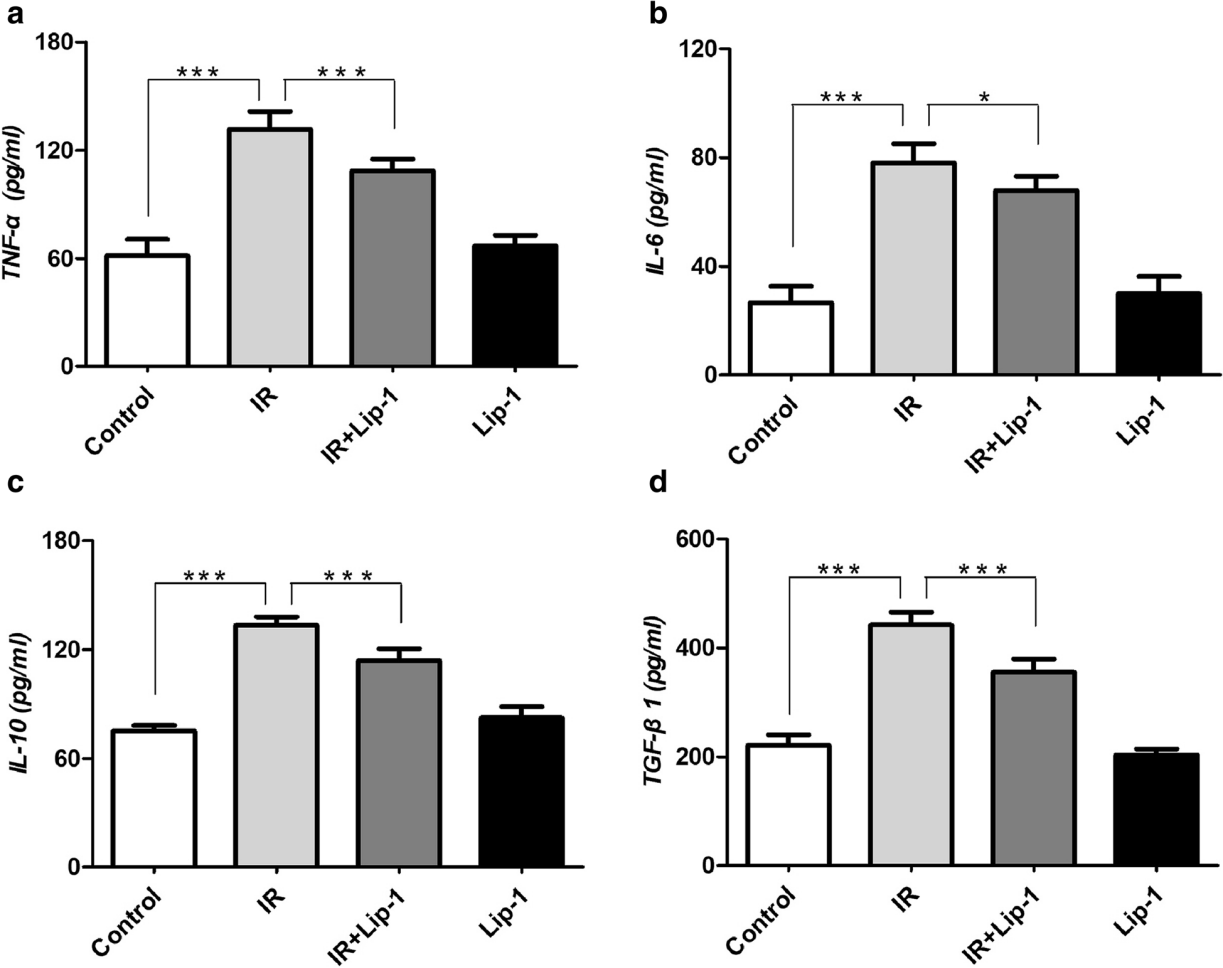
Effects of liproxstatin-1 on serum cytokines following RILF. **a**, **b**, **c**, **d**: The levels of TNF-α, IL-6, IL-10 and TGF-β1 were evaluated by Elisa. (Data shown as mean ± SD, one-way ANOVA followed by a Bonferroni correction, * *P* < 0.05, ** *P* < 0.01, *** *P* < 0.001, *n* = 6)

The production and release of inflammatory cytokines exert a critical role in the pathogenesis of RILF [8, 9]. To determine if the liproxstatin-1 mediated reduction of pulmonary fibrosis was accompanied by a reduction of inflammatory cytokines, serum levels of TNF-α, IL-6, IL-10, and TGF-β1 in each groups of mice were measured by Elisa (Fig. 4a-d). Treatment with the liproxstatin-1 significantly reduced the radiation-induced expression of TNF-α (*p* < 0.001), IL-6 (*p* < 0.05), IL-10 (*p* < 0.001), and TGF-β1 (*p* < 0.001). These data indicated that ferroptosis inhibitor eliminated RILF through down-regulating of TNF-α, IL-6, IL-10, and TGF-β1.

**Fig. 5:**
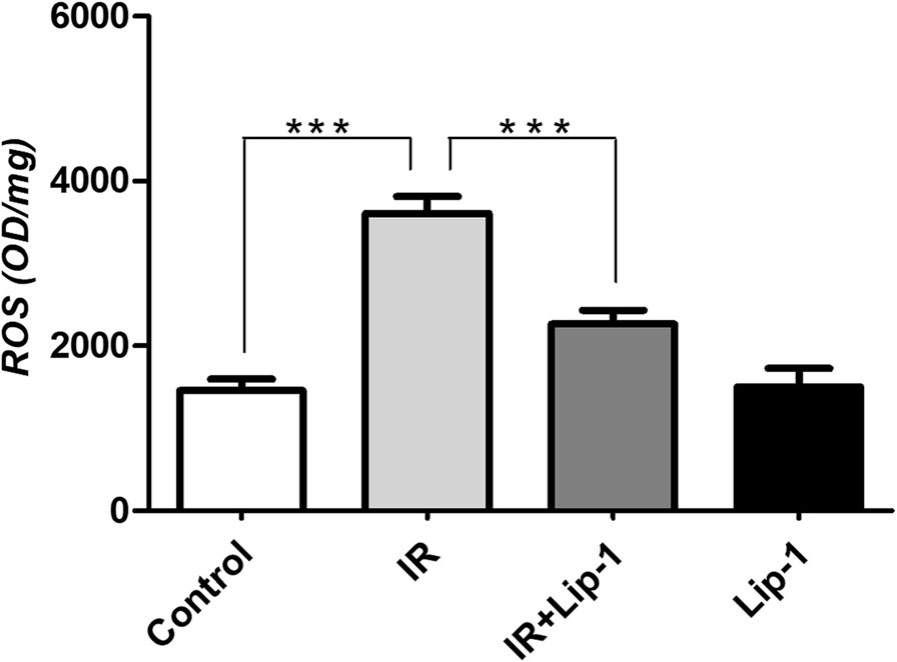
The effect of lipoxstatin-1 on ROS levels in irradiated lungs. (Data shown as mean ± SD, one-way ANOVA followed by a Bonferroni correction, *** *P* < 0.001, *n* = 6)

ROS induced by IR were reported to induce pulmonary injury, inflammation and fibrosis in RILF [10, 11]. ROS-induced oxidative damage is considered to be a critical origin of inflammatory events involved in RILF. ROS is also the major trigger of ferroptosis. In this study, the ROS levels of the irradiated lungs were significantly increased (Fig. 5, *p* < 0.001), after treatment with liproxstatin-1, the levels of ROS were significantly down-regulated (Fig. 5, *p* < 0.001).

### Ferroptosis inhibitor activated Nrf2 signaling in RILF

**Fig. 6:**
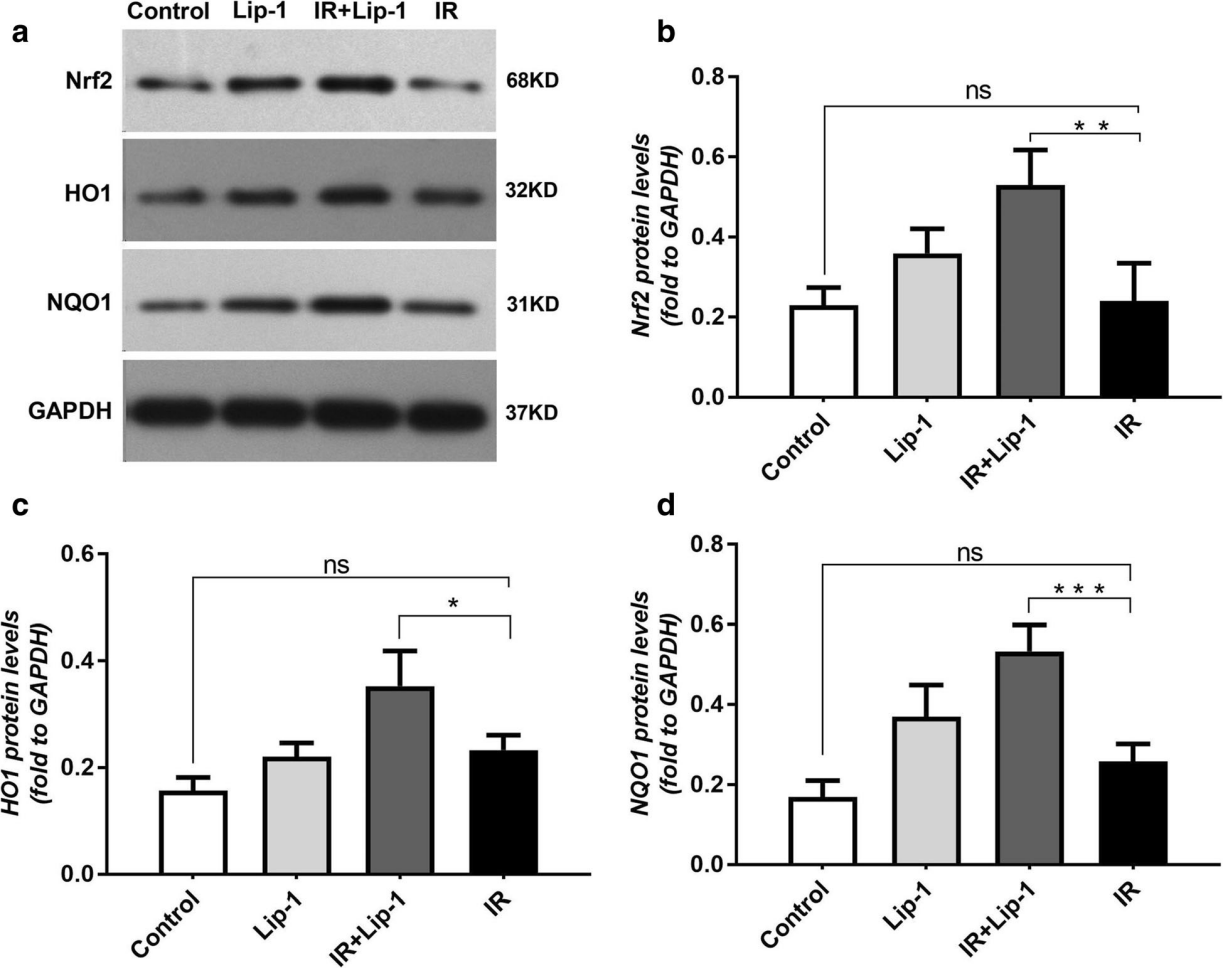
Effects of liproxstain-1 on the proteins levels of Nrf2 pathway following RILF. **a** The expressions of Nrf2, HO1 and NQO1 in lungs were evaluated by western blotting. **b**, **c**, **d** Quantification of Nrf2, HO1 and NQO1 expressions. (Data shown as mean ± SD, one-way ANOVA followed by a Bonferroni correction, ^ns^ Represented no statistical difference. * *P* < 0.05, ** *P* < 0.01, *** *P* < 0.001, *n* = 6)

**Fig. 7:**
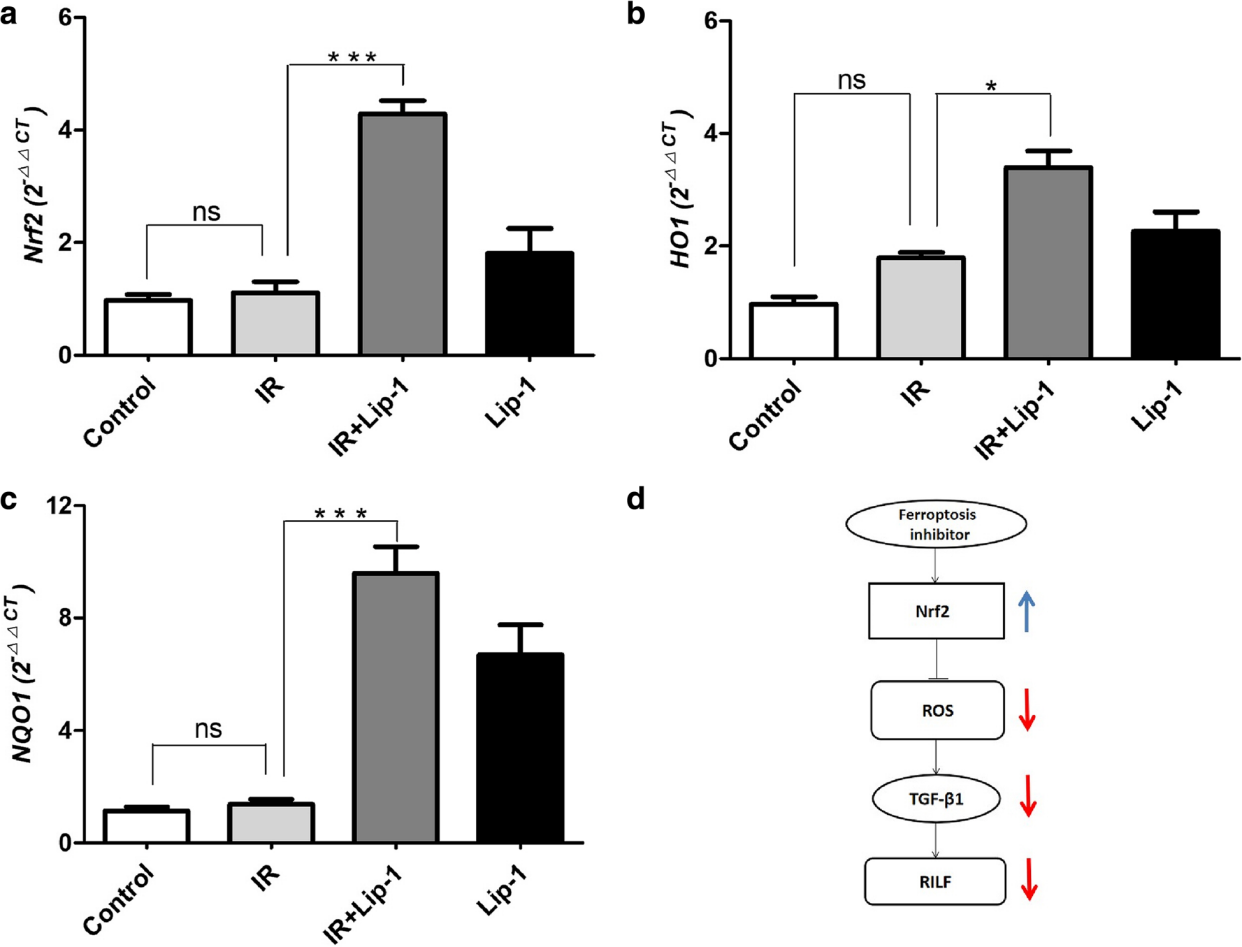
Effects of liproxstain-1 on the mRNA levels of Nrf2 pathway following RILF. **a**, **b**, **c** Quantification of Nrf2, HO1 and NQO1expressions. The mRNA levels of Nrf2, HO1 and NQO1 in lungs were evaluated by real time PCR. **d** The schematic diagram of Nrf2 pathway following RILF. (Data shown as mean ± SD, one-way ANOVA followed by a Bonferroni correction, ^ns^ Represented no statistical difference. * *P* < 0.05, *** *P* < 0.001, *n* = 6)

Activation of the Nrf2 signaling could down-regulates TGF-β1, and alleviates many fibrosis diseases [23–25]. Nrf2 signaling also plays a protective role against ferroptosis in many diseases [26, 27]. HO1 and NQO1 are the vital antioxidant enzymes that protect the cells and tissues from ROS damage in Nrf2 pathway [32]. Suppression of HO1 and NQO1 expression significantly increased ferroptosis in HCC cells [26]. In this study, the protein and mRNA levels of Nrf2, HO1 and NQO1were assessed by western blot analysis (Fig. 6a-d) and Real time PCR analysis (Fig. 7a-c) relatively. We found that treatment with liproxstatin-1 significantly increased the protein levels of Nrf2 (Fig. 6b, *p* < 0.01), HO1 (Fig. 6c, *p* < 0.05) and NQO1 (Fig. 6d, *p* < 0.001) in RILF mice. The analysis of Real time PCR showed liproxstatin-1 also significantly increased the mRNA levels of Nrf2 (Fig. 7a, *p* < 0.001), HO1 (Fig. 7b, *p* < 0.05) and NQO1 (Fig. 7c, *p* < 0.001) in RILF mice. The results suggested that ferroptosis inhibitor activated the Nrf2 signaling in RILF. Taken the results together, the main relationships of ferroptosis inhibitor on Nfr2 pathway in RILF were shown as Fig. 7d.

## Discussion

To date, the mechanisms of RILF are not yet fully understood. Excessive accumulation of ROS and a burst of inflammatory cytokines, especially TGF-β1, play critical roles in the pathogenesis of RILF. Cell death is a key issue in RILF. Traditionally, it is believed that cell death in RILF is apoptosis caused by the direct DNA damage from the radiation [33, 34]. But the radiation not only damages cell DNA, but also induces the production of ROS which can cause inflammation and fibrosis in RILF. Therefore, there may be other types of cell death besides apoptosis in RILF. Ferroptosis is a form of iron-dependent, lipid oxidation-mediated programmed cell death implicated in a wide array of disease pathologies including inflammation, neurodegenration, and ischemia-reperfusion injury [12, 15]. This study was undertaken to explore the role of ferroptosis in RILF and the therapeutic effects and mechanism of ferroptosis inhibitor on RILF.

It is essential to find a potent and stable ferroptosis specific inhibitor for in vivo study. Ferrostatin-1 (Fer-1) is the first generation of ferroptosis inhibitor and it actively suppressed ferroptosis in vitro [35]. However, its in vivo functionality is weak due to plasma and metabolic instability. Liproxstatin-1 is more stable and potent than Fer-1, moreover, it has no interpretation with other classical types of cell death [28]. It was used to suppress ferroptosis in many vivo studies [26–28].

GPX4 is the central regulator of ferroptosis, and the decline of GPX4 is often used as a marker of ferroptosis [12, 14, 28]. In this study, we found that upon RILF, GPX4 was down-regulate obviously, and the ferroptosis inhibitor increased the expression of GPX4. This data supported that the ferroptosis occurred in RILF. Here in RILF model, the administration of liproxstatin-1 inhibited the collagen deposition and reduced the contents of HYP. These results indicated that the ferroptosis played a crucial role in the process of RILF and the ferroptosis inhibitor could reduce the histological alteration in RILF mice.

At the cellular level, radiation could induce a burst of inflammatory and fibrotic cytokines, stimulating the progression of RILF. Among these cytokines, TGF-β1 has been shown to exert a critical role in the pathogenesis and development of RILF [8, 9]. Inhibition of TGF-β1 expression has been speculated to be an effective approach to treat RILF [36, 37]. In this study, liproxstatin-1 treatment lowered the levels of pro-inflammatory cytokines IL-6, TNF-α, IL-10, and TGF-β1 significantly. Ferroptosis inhibitor may results in prohibition of inflammatory cascade in RILF. Lipid peroxidation in ferroptosis may generate signing molecules of inflammation. This result was consistent with the effects of ferroptosis inhibitor which reduced inflammatory cytokines including TGF-β1 in previous studies [16, 17]. The excessive accumulation of ROS induced by IR causes oxidative damage in lung tissues. ROS induced by IR were reported to induce pulmonary injury, inflammation and fibrosis in RILF [10, 11]. ROS-induced oxidative damage is considered to be a critical origin of inflammatory events involved in RILF. ROS is also the major trigger of ferroptosis. Ferroptosis is induced by failure of membrane lipid repair, resulting in the accumulation of ROS on the membrane lipids. Hence, the excessive accumulation of ROS induced by IR may be trigger of the ferroptosis in RILI. GPX4 mainly express in airway epithelial cells. However, collagen/fibrosis are in mouse parenchyma. We consider the reasons might be as follow, the radiation induces the ferroptosis of the airway epithelial cells, which up-regulates the release of inflammatory cytokines including TGF-β1. These inflammatory cytokines cause collagen/fibrosis in lung parenchyma. Our data showed treatment with liproxstatin-1, the levels of ROS were significantly down-regulated in RILF. Together with the results above, our research suggested that ferroptosis inhibitor liproxstatin-1 might eliminate RILF by down-regulating of TGF-β1 via suppression of ROS.

Nrf2 is the central player in the regulation of antioxidant molecules in cells [18]. When intracellular ROS is excessive, the endogenous Nrf2 signaling pathway will be activated. In the radiation-induced lung injury, the radiation not only reduced excessive accumulation of ROS, but also inhibited the over express of endogenous Nrf2 signaling pathway [38]. Our study also found that in RILF, the ROS levels was increased than the control group (Fig. 5), but the expression of Nrf2 was nearly the same as the control group (Fig. 6). These results supported that radiation inhibited the over express of endogenous Nrf2 signaling pathway, although the excessive accumulation of ROS was induced in RILF. The activation of the Nrf2 signaling could down-regulates TGF-β1, and alleviates the fibrosis diseases including RILF [23–25, 39]. Nrf2 plays a key role in the protection of cells and tissues from oxidative damage via up regulation of detoxification enzymes, including glutathione S-transferases, NOQ1, antioxidant enzymes such as HO1, peroxiredoxin1 and cystine transport activity [32]. Nrf2 signaling has also been reported to have a protective role against ferroptosis. In HCC cells, Nrf2 mediated anti-ferroptosis activity depends on the induction of NOQ1, HO1, and FTH1 [26]. The Nrf2 pathway is also involved in the mechanism underlying ferroptosis in head and neck cancer and Parkinson’s disease [17, 35]. In this study, ferroptosis inhibitor activated the Nrf2 pathway in RILF, which up-regulated the levels of HO1 and NQO1, and reduced the damage of ROS. Our results were consistent with the previous studies of Nrf2 on ferroptosis. Taking these data together, our study strongly supported the idea that the ferroptosis inhibitor exerted its inhibitory effects on RILF by down-regulating TGF-β1 expression. Moreover, the ferroptosis inhibitor induced the activation of Nrf2 pathway, which subsequently reduced ROS, leading to down-regulation of TGF-β1 and attenuated RILF in mice. This pathway was shown as Fig. 7d. What’ more, the Nrf2 in the group that mice received lipoxstatin-1 only was also showed a little increase, and the ROS level (Fig. 5) was nearly the same as the control group. We thought there might be some other signings to balance the ROS level in the normal individuals. The level of NOQ1, detoxification enzymes, was also increased obviously in mice treated with lipoxstatin-1 only (Fig. 7c), and this might be related to that Lip-1 could activate the Nrf2 signing [26]. The specific regulatory relationships of genes in Nrf2 pathway on frerroptosis in RILF and the effects of ferroptosis on inflammatory microenvironment of RILF have not been studied intensively. There might be other signings that Lip-1 could activate the NOQ1 directly. A long-term study to identify the roles of Nrf2 in ferroptosis on RILF both in vivo and vitro is required.

## Conclusions

In conclusion, we explored that ferroptosis played a crucial role in RILF, and the ferroptosis inhibitor alleviated RILF via down-regulation of TGF-β1 by the activation of Nrf2 pathway. Ferroptosis inhibitor might be an effective therapy for RILF patients.

## Data Availability

All data used during the current study available from the corresponding author on reasonable request.

## Abbreviations

GPX4: Glutathione peroxidase 4
H&E staining: Hematoxylin eosin staining
HO1: Hemeoxygenase-1
HYP: Hydroxyproline
IL-10: Interleukinn-10
IL-6: Interleukin-6
IR: Ionizing radiation
NQO1: Quinone oxidoreductase 1
Nrf2: Nuclear factor (erythroid-derived 2)-like 2
RILF: Radiation- induced lung fibrosis
ROS: Reactive oxygen species
TGF-β1: Transforming growth factor-β1
TNF-α: Necrosis factor–α
